# Jie-Yu-He-Huan Capsule Ameliorates Anxiety-Like Behaviours in Rats Exposed to Chronic Restraint Stress via the cAMP/PKA/CREB/BDNF Signalling Pathway

**DOI:** 10.1155/2021/1703981

**Published:** 2021-10-04

**Authors:** Xiwen Geng, Hongyun Wu, Zifa Li, Chuanfen Li, Dan Chen, Jiancheng Zong, Zimin Liu, Sheng Wei, Wei Peng

**Affiliations:** ^1^Experimental Centre, Shandong University of Traditional Chinese Medicine, Ji'nan, 250355 Shandong, China; ^2^No. 3 Department of Encephalopathy, Affiliated Hospital of Shandong University of Traditional Chinese Medicine, Ji'nan, 250011 Shandong, China; ^3^College of Physical Education, Shandong Normal University, Ji'nan, 250014 Shandong, China; ^4^Chenland Nutritionals, Inc., Irvine, 92614 CA, USA

## Abstract

Chronic stress is a critical factor in the aetiology of anxiety disorders; however, in the clinic, enduring and preventive measures are not available, and therapeutic drugs are associated with inevitable side effects. Our study established an anxiety rat model using chronic restraint stress (CRS) and assessed these animals using the open-field test, elevated plus-maze test, and light-dark box test. Jie-Yu-He-Huan capsule (JYHH), a Chinese medicine formula, was used as a preventative drug. The HPA axis-mediated release of corticotropin-releasing hormone, adrenocorticotropic hormone, and corticosterone from the hypothalamus was tested. In the hippocampus and prefrontal cortex, concentrations of 5-HT and its metabolite 5-hydroxyindoleacetic acid, as well as monoamine oxidase A, glucocorticoid receptor, and 5-HT1A receptor expression levels, were measured. Furthermore, we examined protein and mRNA expression of cAMP-PKA-CREB-BDNF pathway components. The results showed that JYHH had a significant preventative effect on the anxiety-like behaviour induced by CRS and prevented abnormal changes in the HPA axis and 5-HT system. Furthermore, CRS inhibited the cAMP-PKA-CREB-BDNF pathway, which returned to normal levels following JYHH treatment. This might be the underlying molecular mechanism of the antianxiety effect of JYHH, which could provide a new clinical target for preventative anxiolytic drugs for chronic stress.

## 1. Introduction

Stress is considered a normal physical and psychological reaction to positive or negative situations. Chronic stress is a critical factor in the aetiology of psychiatric diseases, such as anxiety disorders [1], and impacts behavioural, endocrine, and brain functions. A chronic restraint stress (CRS) animal model is commonly used to mimic the pathogenesis and pathophysiology of chronic stress-induced anxiety [2]. Recently, with the continuous acceleration of the pace of life, the incidence of anxiety disorders is increasing. Currently, the first-line treatment of anxiety is benzodiazepines, but their long-term use can cause drug dependence and memory and cognitive impairment, increasing the risk of motor function damage and affecting its clinical application [3]. Traditional Chinese medicine has fewer side effects and strong individualization with high clinical research value [4]. Therefore, it has attracted increasing attention for the treatment of anxiety.

The hypothalamic–pituitary–adrenal (HPA) axis and its response to chronic stress are key endocrine adaptors to stressors [5]. Moreover, overactivated HPA functions are associated with anxiety disorders [6]. In the central nervous system, chronic stress can alter its architecture, molecular profile, and neurochemistry. Stress-induced dysregulation of the HPA axis (especially increased CORT, the final output of the HPA axis) downregulates glucocorticoid receptor (GR) expression in the hippocampus and prefrontal cortex (PFC) as a feedback control mechanism [5, 7].

Dysfunction of the 5-hydroxytryptamine (5-HT) system and neurotransmitter receptor-mediated signal transduction pathways in the brain are strongly correlated with stress-induced emotional and behavioural disorders such as anxiety and depression [8, 9]. Indeed, 5-HT1A receptor (5HT1AR) knockout mice are more anxious than wild-type mice [10]. In the disease pathogenesis, the interaction between the HPA axis and the central 5-HT system is crucial [11]. The therapeutic effects of selective serotonin reuptake inhibitors involve significant changes in the HPA axis [12]. Moreover, antianxiety agents act by regulating some molecules (e.g., GR, CRH, and CORT) [13, 14].

To understand the molecular mechanisms involved in stress-induced anxiety and drug treatments, various studies have explored different components of the HPA axis, including the cyclic adenosine monophosphate- (cAMP-) mediated second messenger cascade. Upregulation of the 5-HT system and stimulation of G-protein coupled receptors, such as 5-HT1AR, might involve cAMP-protein kinase A (PKA) activation [15]. Therefore, cAMP-PKA cascade activation could be translated to the cAMP response element-binding protein (CREB) with a subsequent increase in brain-derived neurotrophic factor (BDNF) expression [16]. More importantly, BDNF participates in HPA axis regulation in CRS-induced emotional disorder [17].

Traditional Chinese medicine can treat anxiety [18–20]. The mechanism is intricate and reportedly involves HPA axis regulation or BDNF [21, 22]. Thus, a rat model with CRS-induced anxiety-like behaviour was established, and behavioural tests were performed. Jie-Yu-He-Huan capsule (JYHH), a Chinese medicine formula, was used as a preventative drug. We hypothesised that JYHH can prevent anxiety-like behaviour by regulating the cAMP-PKA-CREB-BDNF pathway through the HPA axis and 5-HT system. Therefore, concentration or expression of relevant components of the HPA axis, 5-HT system, and cAMP-PKA-CREB-BDNF pathway was examined.

## 2. Methods

### 2.1. Animals, Groups, and Experimental Design

**Figure 1:**
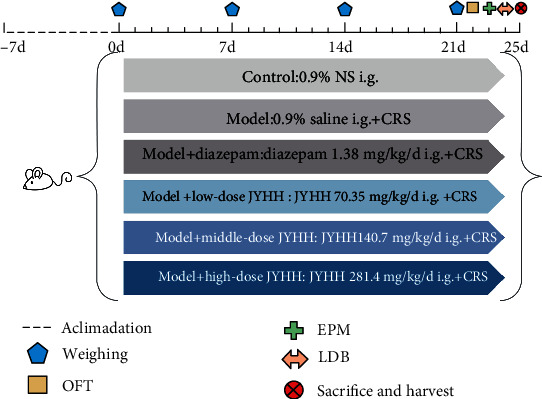
Representation of the overall experimental design and flow chart. i.g.: intragastrically; CRS: chronic restraint stress; JYHH: Jie-Yu-He-Huan capsule; OFT: open field test; EPM: elevated plus maze; LDB: light dark box.

In this study, 6 to 8-week-old male Wistar rats (weighing 140–160 g) were purchased from Vital River Laboratories (Beijing, China) [Laboratory animal production license number: SCXK (Jing)2016-0006]. Animals were acclimated for 1 week and housed at 21 ± 1°C with 55% relative humidity and a 12/12 h light/dark cycle [Laboratory animal use license number: SYXK(Lu)2017-0022]. Food and water were provided *ad libitum*. The experiments were performed in accordance with Guide for The Care and Use of Laboratory Animals approved by the Governing Board of the National Research Council and were approved by the Ethics Review Board of Shandong University of Traditional Chinese Medicine (no. DWSY201908006) [23]. All behavioural experiments were conducted during the dark cycle (09:00 a.m. to 05:00 p.m.) under dim red light conditions [24]. Rats were divided into the following six groups using a randomised-block design according to their weight and the total distance in the OFT: control, model, model+low-dose JYHH (low), model+middle-dose JYHH (middle), model+high-dose JYHH (high), and model+diazepam (diazepam). The overall experimental design and flow chart are demonstrated in Figure 1.

### 2.2. Chronic Restraint Stress

As a noninvasive stimulus, CRS is widely used to study anxiety-related behaviours. According to the daily restraint time and total restraint days, different CRS schemes are used to prepare the models of stress-induced anxiety; they also cause changes in the structure and related molecules of different brain regions of the nervous system. To prepare the stress-induced anxiety model, rats in the low, middle, high, and diazepam groups were subjected to CRS for 21 days. A transparent plastic tube (height, 5 cm; inner diameter, 5.5 cm; and length, 22 cm) was used for restraint stress; the length was adjusted according to the body weight of the rats. In the restraint state, rats were allowed to breathe freely through the evenly distributed vent holes on the plastic tube but not move. The rats were restrained in the tube in their usual home cages, keeping a supine position every day for 6 h (from 9:00 to 15:00) [25–28]. Rats in the control group were transferred to the same room at the same time every day without other treatments.

### 2.3. Extract Preparation and Analysis

The Chinese medicine formula JYHH consists of extracts from Paeoniae Radix Alba, Fructus Gardeniae, Albiziae Flos, and Moutan Cortex in the following respective proportions: 52.1%, 27.0%, 18.7%, and 2.2%. The extract of each component was prepared as follows. Paeoniae Radix Alba was added to an 8-fold volume of 70% ethanol and heated for 2 h using the reflux extraction method. After filtration, a 6-fold volume of deionised water was added to the sediment, and heating reflux extraction was performed twice for 1 h each time. The ethanol and water extracts were mixed and concentrated to a thick paste using a rotary evaporator (DLSB-6/10, Shanghai YaRong). After drying at 60°C under reduced pressure (DZF-6050, Shanghai YiHeng), the extract from Paeoniae Radix Alba was pulverised (GLG-2777, Shanghai Ezaki Glico Foods) and passed through an 80-mesh sieve. Fructus Gardeniae was crushed preliminarily and extracted by heating reflux three times (2 h each time) with 6-fold, 5-fold, and 4-fold volumes of 70% ethanol, respectively. After filtration, the extracts were concentrated and dried at 60°C under reduced pressure, pulverised, and passed through an 80-mesh sieve. Albiziae Flos was crushed preliminarily and extracted by heating reflux twice (2 h each time) with an 8-fold volume of 70% ethanol. After filtration, the extract was concentrated, dried at 60°C under reduced pressure, pulverised, and sieved as with the other extracts. Moutan Cortex was crushed preliminarily and added to a 14-fold volume of pure water. After letting stand for 24 h, a 9-fold volume of the component was extracted by heating. Then, the final extract was obtained by crystallisation, filtration, and drying after letting stand at 4°C for 24 h.

Analyses of the extracts were performed using high-performance liquid chromatography (HPLC, Agilent Technologies 1260 Infinity). JYHH (0.5 g) was suspended in 25 mL of methanol and ultrasonicated for 30 min at 20°C. After passing through a 0.45 *μ*m Millipore filter, the sample was transferred to an autosampler, and 10 *μ*L was injected for analysis. Analyte separation was performed with a C18 column (4.6 × 250 mm, 5 *μ*m, Agilent 5 TC) at 30°C. Mobile phase A was 100% acetonitrile, and mobile phase B was a 0.1% aqueous potassium phosphate solution. The gradient program was set as follows: 5% A, 0–15 min; 14% A, 15–30 min; 15% A, 30–35 min; 20% A, 35–50 min; 35% A, 50–70 min; 95% A, 70–81 min; and 5%, 81–90 min. The flow rate was 1 mL/min, and the detection wavelength was 300 nm.

### 2.4. Drug Treatment

Drug treatment was carried out daily before CRS for 21 days. Rats in the diazepam group received 1.38 mg/kg diazepam (batch no. 20180402, Beijing Yimin Pharmaceutical, Beijing, China) dissolved in 10 mL 0.9% saline, intragastrically [29]. Rats in the low, middle, and high dose groups were administered JYHH dissolved in 10 mL 0.9% saline, intragastrically, at doses of 70.35, 140.7, and 281.4 mg/kg, respectively. The dosage of JYHH in rats was converted based on the equivalent dose coefficient of human clinical dosage according to the “Pharmacological Experiment Methodology Third Edition” [30]. Rats in the control group received the same volume of 0.9% saline at the same times.

### 2.5. Open Field Test

On the day after the final drug treatment and CRS (day 22 of the experiment schedule), the OFT was carried out with a square apparatus (100 × 100 cm) in which the arena was divided into nine equal squares. Each rat was placed in the centre square and allowed to roam freely for 6 min. The trajectory was recorded by a camera, and the total distance, centre area distance, and time in the centre area were recorded using the XR-Super Maze tracking system (Shanghai Xinsoft Information Technology, Shanghai, China) [24]. After each trial, the apparatus was carefully cleaned with 70% ethanol.

### 2.6. Elevated plus Maze Test

On day 23 of the experiment, the EPM test was performed with a polypropylene plastic cruciform apparatus consisting of two open arms (10 × 50 cm), two closed arms (10 × 50 cm), and a centre platform (10 × 10 cm). The apparatus was elevated 76 cm above the floor, and rats were placed on the centre platform with their head towards the open arm. Rat behaviour was recorded for 5 min using the XR-Super Maze tracking system, and the open-arm entry times (OEs), closed arm entry times (CEs), time in the open arm (OT), and time in the closed arm (CT) were analysed. The OE percentage was calculated as OE/(OE + CE) × 100, and the OT percentage was calculated as OT/(OT + CT) × 100 [31].

### 2.7. Light-Dark Box Test

On day 24 of the experiment schedule, the LDB test was performed with a box consisting of dark and light chambers (25 × 25 × 30 cm) separated by a 6.5 × 6.5 cm door. The test started with the placement of the rats at the centre of the light chamber and lasted for 5 min. The XR-Super Maze tracking system was used to record the behaviour and analyse the total distance, light area distance, time in the light area, and light area entries [32].

### 2.8. Analysis of HPA Axis Hormones in Serum

After the behavioural tests, rats were sacrificed with an overdose of 2% pentobarbital sodium and 5 mL of peripheral blood were collected. After centrifugation at 3000 × *g* at 4°C for 15 min, the plasma was separated for analyses of CRH, ACTH, and CORT using enzyme-linked immunosorbent assay (ELISA) kits (CSB-E08038r, CSB-06875r, CSB-E07014r, respectively; Cusabio, Wuhan, China). The optical density at 450 nm was measured using a microplate reader. Each sample was subjected to three duplicate tests to avoid any errors.

### 2.9. Analysis of 5-HT and 5-HIAA

Rat brains were removed rapidly, and the hippocampus and PFC were isolated on ice and frozen at −80°C. Concentrations of 5-HT and 5-HIAA in each group were quantified using HPLC-tandem mass spectrometry (Shimadzu LC20AD-API 3200MD TRAP). The brain tissue was homogenised in precooled pure water and centrifuged at 4°C for 1 min at 13,200 × *g*. Then, the supernatant was added to a 3-fold volume of precipitant (methanol/acetonitrile = 1 : 1) and centrifuged at 4°C for 4 min at 13,200 rpm. The MSLab C18 column (100 × 4.6 × 3 *μ*m) was used at 50°C. A 3 *μ*L sample was injected with water phase A (pure water containing 0.1% formic acid) and organic phase B (methanol containing 0.1% formic acid). To identify 5-HT, the gradient program was set as follows: 98% A, 0.0–0.5 min; 80% A, 0.5–2.5 min; 30% A, 2.5–4.5 min; 0% A, 4.5–6.5 min; and 98% A, 6.5–8.0 min. The flow rate was 0.6 mL/min. To identify 5-HIAA, the gradient program was set as follows: 85% A, 0.0–1.0 min; 30% A, 1.0–3.5 min; 0% A, 3.5–5.0 min; and 85% A, 5.0–7.5 min. The flow rate was 0.8 mL/min.

### 2.10. Measurements of cAMP and PKA

Brain tissue was homogenised in precooled 0.01 M phosphate-buffered saline (pH = 7.4) and centrifuged at 13,000 × *g* at 4°C for 10 min, and the supernatant was collected. The concentrations of cAMP and PKA were measured using ELISA kits (JL20768 and JL13535, respectively; JiangLai Bio, Shanghai, China). The optical density at 450 nm was measured using a microplate reader. Each sample was subjected to three duplicate tests to avoid any errors.

### 2.11. Western Blots

The hippocampus and PFC tissues were homogenised in extraction buffer (R0010; Solarbio Science & Technology, Beijing, China) and ultrasonicated for 10 min. After centrifugation at 12,000 rpm (4°C) for 30 min, the supernatant was collected and the protein concentration was determined with a bicinchoninic acid protein assay kit (PC0020; Solarbio Science & Technology). Samples (20 *μ*L) were loaded onto a 10% sodium dodecyl sulphate–polyacrylamide gel and separated by electrophoresis. Proteins were then transferred electrophoretically to a polyvinylidene difluoride membrane and blocked in 5% nonfat milk for 1 h. The membranes were incubated with the following primary antibodies from ABclonal Biotechnology (Wuhan, China): 1 : 1000, rabbit anti-CREB, A10826; 1 : 1000 rabbit anti-phosphorylated- (p-) CREB, AP0019; 1 : 1000 rabbit anti-BDNF, A4873; 1 : 10,000 mouse anti-GAPDH, AC002; 1 : 2000 rabbit anti-*β*-tubulin, AC008; 1 : 1000 rabbit anti-MAO-A, A4105; 1 : 1000 rabbit anti-GR, A19583; and 1 : 1000 rabbit anti-5-HT1AR, A2801. The secondary antibodies were 1 : 5000 horseradish peroxidase-conjugated goat anti-rabbit IgG (H+L) (ABclonal, AS014) or goat anti-mouse IgG (H+L) (ABclonal, AS003). Protein bands were visualised using an enhanced chemiluminescence reagent (PE0010, Solarbio Science & Technology). The optical density value was calculated using the ImageJ software.

### 2.12. Reverse Transcription Real-Time Quantitative Polymerase Chain Reaction (RT-qPCR)

Total RNA was extracted from the hippocampus and PFC tissues using TRIzol reagent (RC101; Vazyme, Nanjing, China) and then reverse transcribed to cDNA using the PrimeScript RT reagent kit (TaKaRa Bio, Shiga, Japan). RT-qPCR was performed using the Roche LightCycler 480 Real-Time PCR System with the SYBR Green qPCR Master Mix (Excell Biotech, Clearwater, FL, USA). The RT-qPCR experiments for each sample were repeated thrice to eliminate errors. The 2^−*ΔΔ*Ct^ method was used for data analysis. *β*-Actin was used as the internal reference. Primers were synthesised by Sangon Biotech (Shanghai, China), and the sequences were as follows:

- BDNF forward: 5′-TGGAACTCGCAATGCCGAACTAC-3′; BDNF reverse: 5′-TCCTTATGAACCGCCAGCCAATTC-3′
- CREB forward: 5′-GGAGCAGACAACCAGCAGAGTG-3′; CREB reverse: 5′-GGCATGGATACCTGGGCTAATGTG-3′
- MAO-A forward: 5′-GACACGCTCAGGAATGGGACAAG-3′; MAO-A reverse: 5′-ACAGGAACCACAGGGCAGATACC-3′
- 5-HT1AR forward: 5′-AGGACCACGGCTACACCATCTAC-3′; 5-HT1AR reverse: 5′-CTGACAGTCTTGCGGATTCGGAAG-3′
- GR forward: 5′-AAGGCGATACCAGGCTTCAGAAAC-3′; GR reverse: 5′-ATGATCTCCAACCCAGGGCAAATG-3′
- *β*-Actin forward: 5′-CTGAGAGGGAAATCGTGCGTGAC-3′; *β*-actin reverse: 5′-AGGAAGAGGATGCGGCAGTGG-3′

### 2.13. Statistical Analyses

Data analyses were performed using the Prism version 8.0.2 software (GraphPad, La Jolla, CA, USA). The mean ± standard error of the mean was used to express data. The data were tested for normality (Kolmogorov–Smirnov test) and homoscedasticity (Levene's test) before being analysed using parametric test. Exceptional data exceeding the mean ± 2 × standard deviation were removed using the modified Layida method [33]. Differences between two groups were compared using an unpaired *t*-test [34]. The significance level was set at *p* < 0.05.

## 3. Results

### 3.1. Extract Analyses

**Figure 2:**
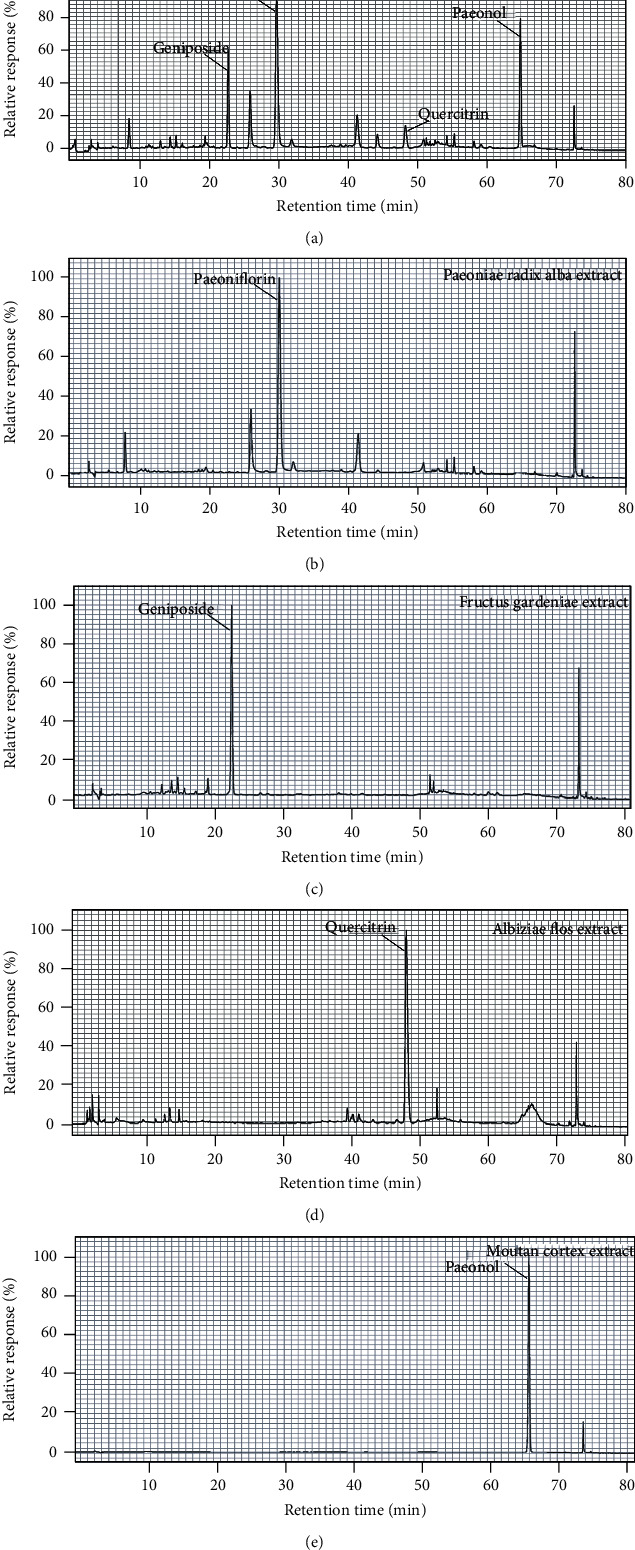
Analyses of the Jie-Yu-He-Huan capsule, Paeoniae Radix Alba, Fructus Gardeniae, Albiziae Flos, and Moutan Cortex extracts.

According to the analytical results in Figure 2, the highest contents of Paeoniae Radix Alba, Fructus Gardeniae, Albiziae Flos, and Moutan Cortex extracts were paeoniflorin (22.7%), geniposide (16.7%), quercitrin (5.2%), and paeonol (100%), respectively. The Chinese medicine formula, JYHH, used as the treatment drug, consisted of extracts from these four herbs. The contents of the four dominating constituents in JYHH were paeoniflorin (12.2%, retention time = 29.8 min), geniposide (4.7%, retention time = 22.8 min), quercitrin (1.1%, retention time = 48.3 min), and paeonol (2.4%, retention time = 64.9 min).

### 3.2. Behavioural Tests

**Figure 3:**
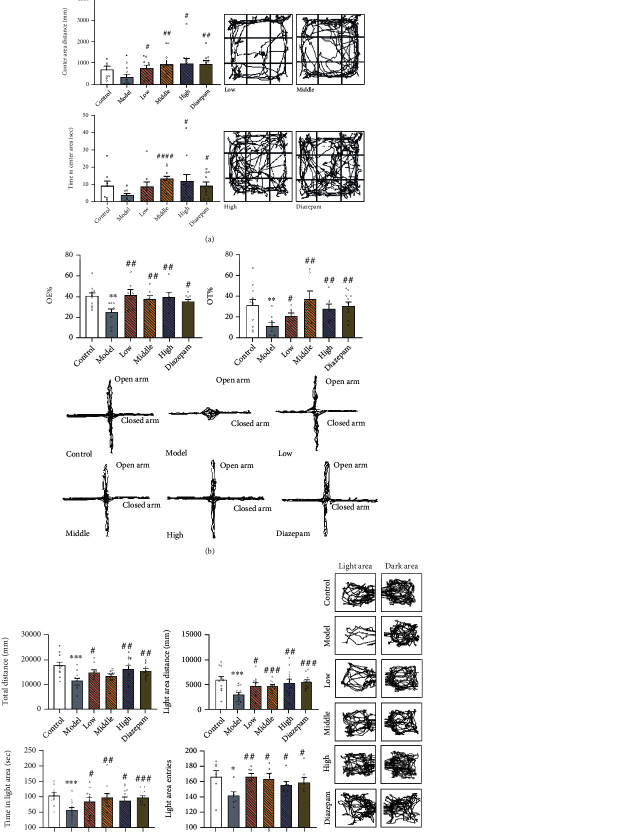
(a) Treatment with Jie-Yu-He-Huan capsule prevents altered behaviour in the open field test caused by chronic restraint stress. ^∗^*p* < 0.05 compared to the control group, ^#^*p* < 0.05 compared to the model group, ^##^*p* < 0.01 compared to the model group, and ^####^*p* < 0.0001 compared to the model group (unpaired *t*-test). (b) Treatment with Jie-Yu-He-Huan capsule prevents altered behaviour in the elevated plus maze test caused by chronic restraint stress. ^∗∗^*p* < 0.01 compared to the control group, ^#^*p* < 0.05 compared to the model group, and ^##^*p* < 0.01 compared to the model group (unpaired *t*-test). (c) Treatment with Jie-Yu-He-Huan capsule prevents altered behaviour in the light-dark box test caused by chronic restraint stress. ^∗^*p* < 0.05 compared to the control group, ^∗∗∗^*p* < 0.001 compared to the control group, ^#^*p* < 0.05 compared to the model group, ^##^*p* < 0.01 compared to the model group, ^###^*p* < 0.001 compared to the model group, and ^####^*p* < 0.0001 compared to the model group (unpaired *t*-test).

In the OFT results (Figure 3(a)), CRS induced a significant decrease in the centre area distance compared to that in the control group (*p* = 0.0448). This was prevented by the low (*p* = 0.0202), middle (*p* = 0.0061), and high (*p* = 0.0104) doses of JYHH and diazepam (*p* = 0.0025). Compared to that in the model group, the middle and high doses of JYHH and diazepam increased the total distance (*p* = 0.0419, *p* = 0.0319, and *p* = 0.0088, respectively) and time in the centre area (*p* < 0.0001, *p* = 0.0325, and *p* = 0.0140, respectively). In the EPM results (Figure 3(b)), rats in the CRS model group showed lower OE (*p* = 0.0015) and OT (*p* = 0.0069) percentages compared to those in the control group, which was prevented by JYHH (low-dose *p* = 0.0049 and 0.0253; middle-dose *p* = 0.0091 and 0.0031; and high-dose *p* = 0.0086 and 0.0065) and diazepam (*p* = 0.0108 and 0.0011).

In the LDB test (Figure 3(c)), CRS induced a decrease in total distance (*p* = 0.0004), light area distance (*p* = 0.0007), time spent in the light area (*p* = 0.0005), and light area entries (*p* = 0.0112). Following treatment with low and high doses of JYHH and diazepam, these alterations were reverted to normal levels (low-dose *p* = 0.0184, 0.0163, 0.0480, and 0.0014; high-dose *p* = 0.0033, 0.0056, 0.0178, and 0.0270; and diazepam *p* = 0.0014, <0.0001, 0.0004, and 0.0313, respectively). Although the middle dose of JYHH did not show any effect on total distance, it increased the light area distance (*p* = 0.0008), time in the light area (*p* = 0.0037), and light area entries (*p* = 0.0128). These behavioural results showing anxiety-like behaviour indicated that we established a successful and reliable anxiety model using CRS, and JYHH treatment showed an anxiolytic effect similar to that of diazepam.

### 3.3. HPA Axis Hormones in Serum

**Figure 4:**
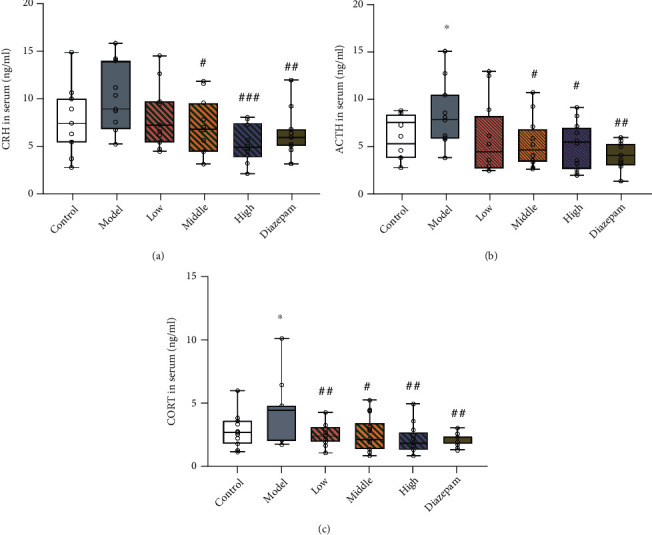
Treatment with Jie-Yu-He-Huan capsule prevents alterations in the hypothalamic–pituitary–adrenal axis hormone concentrations in serum caused by chronic restraint stress. (a) Corticotropin-releasing hormone (CRH) levels. (b) Adrenocorticotropic (ACTH) hormone levels. (c) Corticosterone (CORT) levels. ^∗^*p* < 0.05 compared to the control group, ^#^*p* < 0.05 compared to the model group, ^##^*p* < 0.01 compared to the model group, and ^###^*p* < 0.001 compared to the model group (unpaired *t*-test). The bars show min to max of data, and the small circles represent individual data points.

HPA axis hormones in serum (CRH, ACTH, and CORT) were detected in rats of the different groups. As shown in Figure 4, the concentrations of ACTH (*p* = 0.0427) and CORT (*p* = 0.0450) were significantly higher in the CRS model group than in the control group. Compared to that in the model group, the low dose of JYHH decreased the CORT concentration in the serum (*p* = 0.0093). Rats in the middle- and high-dose groups and the diazepam group had lower CRH (middle-dose *p* = 0.0274, high-dose *p* = 0.0007, and diazepam *p* = 0.0053, respectively), ACTH (middle-dose *p* = 0.0227, high-dose *p* = 0.0139, and diazepam *p* = 0.0011), and CORT (middle-dose *p* = 0.0146, high-dose *p* = 0.0036, and diazepam *p* = 0.0021) concentrations. These results indicate that JYHH treatment could prevent the altered HPA axis hormone concentrations in serum caused by CRS, similar to the effect of diazepam.

### 3.4. Levels of 5-HT and 5-HIAA

**Figure 5:**
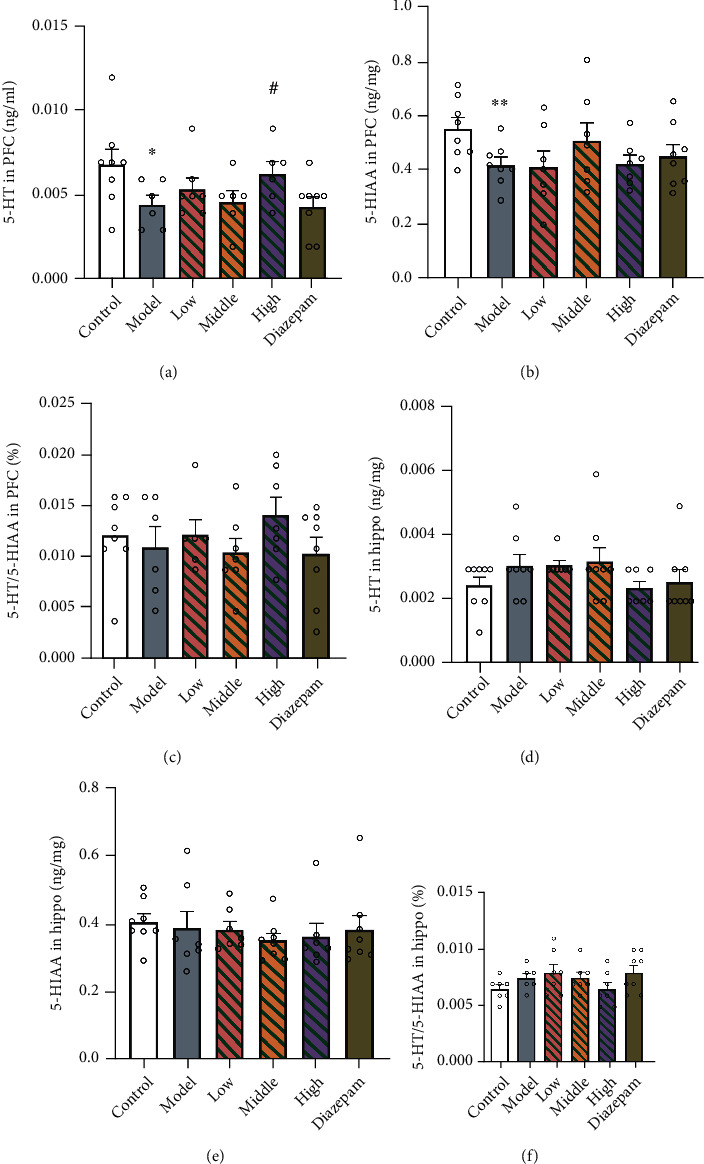
Treatment with Jie-Yu-He-Huan capsule prevents alterations in the 5-hydroxytryptamine (5-HT) and 5-hydroxyindoleacetic acid (5-HIAA) concentrations caused by chronic restraint stress. (a) 5-HT level in the prefrontal cortex (PFC). (b) 5-HIAA level in the PFC. (c) 5-HT/5-HIAA ratio in the PFC. (d) 5-HT level in the hippocampus. (e) 5-HIAA level in the hippocampus. (f) 5-HT/5-HIAA ratio in the hippocampus. ^∗^*p* < 0.05 compared to the control group, ^∗∗^*p* < 0.01 compared to the control group, and ^#^*p* < 0.05 compared to the model group (unpaired *t*-test). Data are expressed as the means ± SEs. The small circles represent individual data points.

An analysis of the PFC and hippocampus tissues (Figure 5) showed that CRS decreased the 5-HT (*p* = 0.0326) and 5-HIAA (*p* = 0.0069) concentrations in the PFC and that high-dose JYHH treatment blocked the effect of CRS on 5-HT (*p* = 0.0358). In the hippocampus, there were no significant alterations in 5-HT and 5-HIAA levels among the different groups.

### 3.5. Expression of GR, 5-HT1AR, and MAO-A

**Figure 6:**
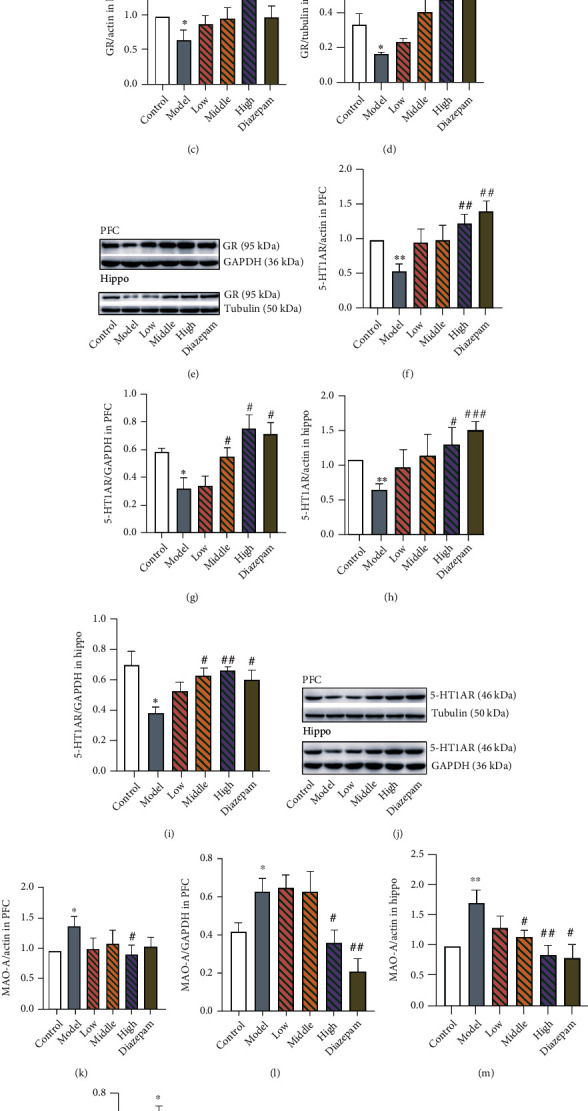
High-dose Jie-Yu-He-Huan capsule treatment has a therapeutic effect on altered glucocorticoid receptor (GR), 5-hydroxytryptamine 1A receptor (5-HT1AR), and monoamine oxidase A (MAO-A) expression caused by chronic restraint stress. (a) GR mRNA expression in the prefrontal cortex (PFC). (b) GR protein expression in the PFC. (c) GR mRNA expression in the hippocampus. (d) GR protein expression in the hippocampus. (e) Representative western blot protein bands of GR expression exhibiting the differences among groups. (f) 5-HT1AR mRNA expression in the PFC. (g) 5-HT1AR protein expression in the PFC. (h) 5-HT1AR mRNA expression in the hippocampus. (i) 5-HT1AR protein expression in the hippocampus. (j) Representative western blot protein bands of 5-HT1AR expression showing the differences among groups. (k) MAO-A mRNA expression in the PFC. (l) MAO-A protein expression in the PFC. (m) MAO-A mRNA expression in the hippocampus. (n) MAO-A protein expression in the hippocampus. (o) Representative western blot protein bands of MAO-A expression showing the differences among groups. ^∗^*p* < 0.05 compared to the control group, ^∗∗^*p* < 0.01 compared to the control group, ^#^*p* < 0.05 compared to the model group, ^##^*p* < 0.01 compared to the model group, and ^###^*p* < 0.001 compared to the model group (*n* = 3 in each group, unpaired *t*-test). Data are expressed as the means ± SEs. The small circles represent individual data points.

In the PFC and hippocampus, CRS induced a significant decrease in GR protein (PFC, *p* = 0.0087; hippocampus, *p* = 0.0300) and mRNA (PFC, *p* = 0.0141; hippocampus, *p* = 0.0306) expression (Figures 6(a)–6(e)). The high dose of JYHH prevented all GR alterations (PFC mRNA, *p* = 0.0118; PFC protein, *p* = 0.0193; hippocampus mRNA, *p* = 0.0202; and hippocampus protein, *p* = 0.0333). The middle dose of JYHH increased GR protein expression in the hippocampus (*p* = 0.0333). In addition, diazepam treatment prevented the GR mRNA (*p* = 0.0297) and protein (*p* = 0.0141) expression changes in the PFC and protein expression changes in the hippocampus (*p* = 0.0462).

Decreased expression of 5-HT1AR was observed in the model compared to that in the control group (Figures 6(f)–6(j): PFC mRNA, *p* = 0.0040; PFC protein, *p* = 0.0145; hippocampus mRNA, *p* = 0.0010; and hippocampus protein, *p* = 0.0168), which were all prevented by high-dose JYHH treatment (PFC mRNA, *p* = 0.0052; PFC protein, *p* = 0.0116; hippocampus mRNA, *p* = 0.0183; and hippocampus protein, *p* = 0.0018) or diazepam (PFC mRNA, *p* = 0.0025; PFC protein, *p* = 0.0116; hippocampus mRNA, *p* = 0.0007; and hippocampus protein, *p* = 0.0181). In addition, the middle dose of JYHH also increased 5-HT1AR protein expression in the PFC (*p* = 0.0428) and hippocampus (*p* = 0.0112).

In the CRS model group, MAO-A mRNA (PFC, *p* = 0.0167; hippocampus, *p* = 0.0081) and protein (PFC, *p* = 0.0402; hippocampus, *p* = 0.0289) expression was higher than that in the control group (Figures 6(k) and 6(l)). The high dose of JYHH maintained normal levels after CRS (PFC mRNA, *p* = 0.0362; PFC protein, *p* = 0.0281; hippocampus mRNA, *p* = 0.0088; and hippocampus protein, *p* = 0.0122). In the diazepam group, MAO-A protein (*p* = 0.0067) and mRNA (*p* = 0.0125) in the PFC and protein (*p* = 0.0030) in the hippocampus were lower than levels in the model animals. The middle dose of JYHH prevented changes in the MAO-A mRNA (*p* = 0.0163) and protein (*p* = 0.0480) levels in the hippocampus. These results indicate that high-dose JYHH treatment has a significant protective effect on the decreases in GR and 5-HT1AR and increases in MAO-A expression associated with CRS-induced anxiety. Importantly, JYHH showed a significant therapeutic advantage over the first-line anxiolytic drug diazepam, as it reversed some alterations that diazepam failed to restore.

### 3.6. Effects on the cAMP-PKA-CREB-BDNF Pathway

**Figure 7:**
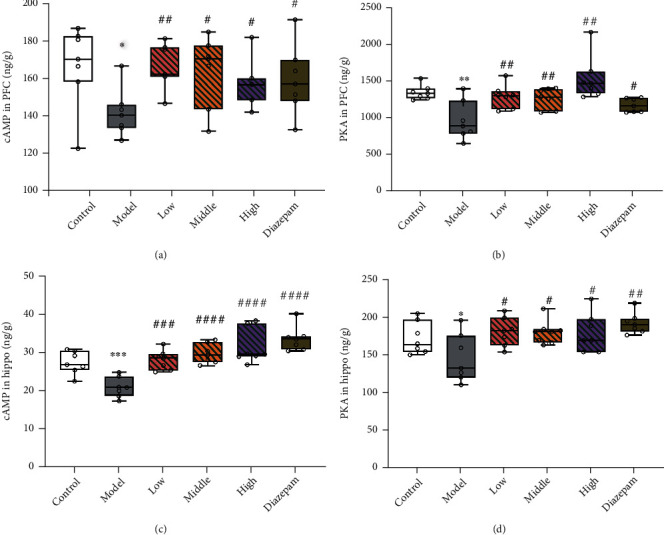
Treatment with Jie-Yu-He-Huan capsule prevents alterations in the cyclic adenosine monophosphate (cAMP) and cAMP-protein kinase A (PKA) concentrations caused by chronic restraint stress. (a) cAMP concentrations in the prefrontal cortex (PFC). (b) PKA concentrations in the PFC. (c) cAMP concentrations in the hippocampus (Hippo). (d) PKA concentrations in the Hippo. ^∗^*p* < 0.05 compared to the control group, ^∗∗^*p* < 0.01 compared to the control group, ^∗∗∗^*p* < 0.001 compared to the control group, ^#^*p* < 0.05 compared to the model group, ^##^*p* < 0.01 compared to the model group, and ^####^*p* < 0.0001 compared to the model group (unpaired *t*-test). The bars show min to max of data, and the small circles represent individual data points.

**Figure 8:**
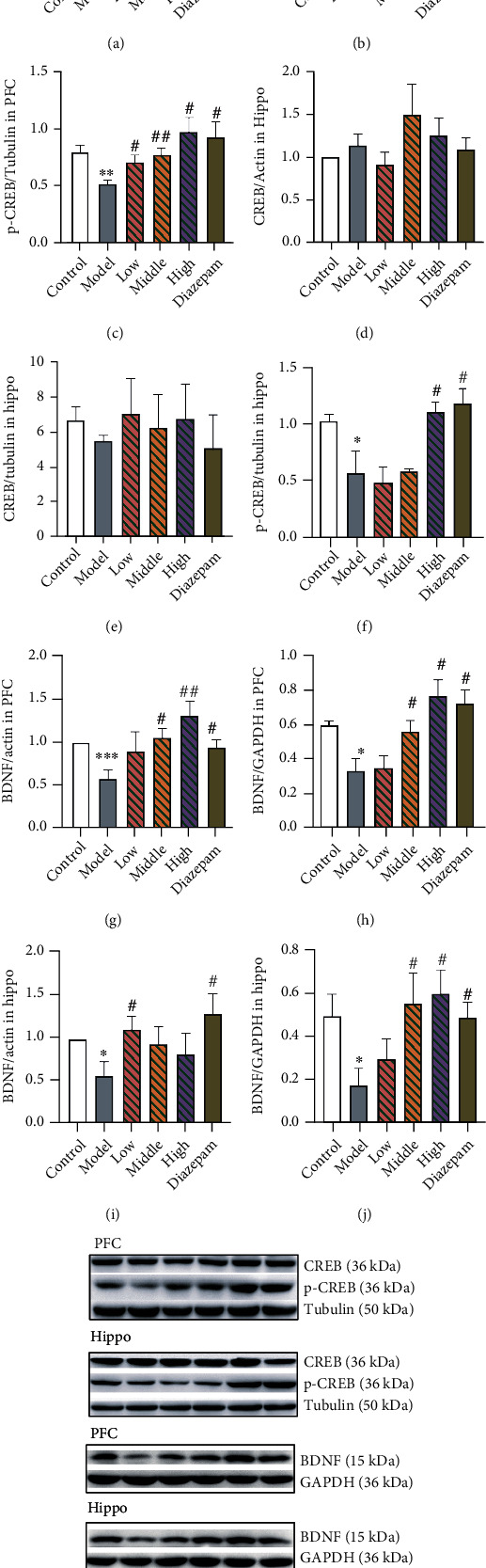
Treatment with Jie-Yu-He-Huan capsule prevents alterations of cyclic adenosine monophosphate response element-binding protein (CREB), phosphorylated- (p-) CREB, and brain-derived neurotrophic factor (BDNF) caused by chronic restraint stress. (a) CREB mRNA expression in the prefrontal cortex (PFC). (b) CREB protein expression in the PFC. (c) Phosphorylated- (p-) CREB protein expression in the PFC. (d) CREB mRNA expression in the hippocampus (Hippo). (e) CREB protein expression in the Hippo. (f) p-CREB protein expression in the Hippo. (g) BDNF mRNA expression in the PFC. (h) BDNF protein expression in the PFC. (i) BDNF mRNA expression in the Hippo. (j) BDNF protein expression in the Hippo. (k) Representative western blot protein bands of CREB, p-CREB, and BDNF expression showing differences among groups. ^∗^*p* < 0.05 compared to the control group, ^∗∗^*p* < 0.01 compared to the control group, ^∗∗∗^*p* < 0.001 compared to the control group, ^#^*p* < 0.05 compared to the model group, and ^##^*p* < 0.01 compared to the model group (*n* = 3 in each group, unpaired *t*-test). Data are expressed as the means ± SEs. The small circles represent individual data points.

Based on the aforementioned observations, we analysed expression levels of components of the cAMP-PKA-CREB-BDNF pathway to explore the molecular mechanism underlying the anxiolytic effect of JYHH. As shown in Figure 7, decreased concentrations of cAMP and PKA were observed in the PFC (cAMP, *p* = 0.0112; PKA, *p* = 0.0017) and hippocampus (cAMP, *p* = 0.0005; PKA, *p* = 0.0437) in CRS-model rats. JYHH or diazepam significantly blocked these alterations (cAMP in the PFC: low-dose JYHH, *p* = 0.0014; middle-dose, *p* = 0.0128; high-dose, *p* = 0.0270; diazepam, *p* = 0.0313; PKA in the PFC: low-dose, JYHH *p* = 0.0071; middle-dose, *p* = 0.0091; high-dose, *p* = 0.0012; diazepam, *p* = 0.0333; cAMP in the hippocampus: low-dose, JYHH *p* = 0.0001; middle-dose, *p* < 0.0001; high-dose, *p* < 0.0001; diazepam, *p* < 0.0001; PKA in the hippocampus: low-dose, JYHH *p* = 0.0142; middle-dose, *p* = 0.0103; high-dose, *p* = 0.0245; diazepam, *p* = 0.0021). In the PFC and hippocampus of CRS-induced anxiety-model rats, there was an obvious decrease in p-CREB (PFC, *p* = 0.0081; hippocampus, *p* = 0.0432) and BDNF (mRNA expression: PFC, *p* = 0.0003 and hippocampus, *p* = 0.0234; protein expression: PFC, *p* = 0.0145 and hippocampus, *p* = 0.0338) (Figure 8). In the PFC, the *CREB* mRNA level was also lower in the model group than in the control group (*p* = 0.0014). This change was prevented by JYHH (low-dose, *p* = 0.0110; middle-dose, *p* = 0.0153; high-dose, *p* = 0.0010) or diazepam (*p* = 0.0084) treatment. However, there were no detected differences in CREB protein expression among the groups in the PFC or hippocampus. Regarding the altered p-CREB in the PFC, JYHH (low-dose, *p* = 0.0296; middle-dose, *p* = 0.0075; high-dose, *p* = 0.0139) or diazepam (*p* = 0.0206) treatment maintained normal levels. However, in the hippocampus, only the high dose of JYHH (*p* = 0.0318) or diazepam (*p* = 0.0312) prevented the decrease in p-CREB. Rats in the diazepam treatment group exhibited higher mRNA (PFC, *p* = 0.0195; hippocampus, *p* = 0.0246) and protein (PFC, *p* = 0.0116; hippocampus, *p* = 0.0206) expression levels of BDNF than the model group. In addition, a low dose of JYHH prevented the decrease in *BDNF* mRNA expression in the hippocampus (*p* = 0.0278). Changes in BDNF mRNA (middle-dose, *p* = 0.0104; high-dose, *p* = 0.0049) and protein (middle-dose, *p* = 0.0428; high-dose, *p* = 0.0016) expression in the PFC, as well as protein (middle-dose, *p* = 0.0392; high-dose, *p* = 0.0167) expression in the hippocampus, could be prevented by the middle and high doses of JYHH.

## 4. Discussion

JYHH is derived from a well-known Chinese medicine classic “Yi Chun Sheng Yi” written by Fei Boxiong (1800-1879). It is mainly used for the treatment of liver-qi stagnation and stagnation fire caused by long-term liver-qi depression. It is clinically used to treat generalized anxiety disorder. The total effective rate of the self-developed symptom scale treatment group is better than that of the control group, showing its advantages, which include fast onset, safe, effective, fewer side effects, and high compliance. The current study demonstrated a significant anxiolytic effect of JYHH. This formula contains extracts from Paeoniae Radix Alba, Fructus Gardeniae, Albiziae Flos, and Moutan Cortex, in which the highest contents were paeoniflorin, geniposide, quercitrin, and paeonol, respectively (Figure 2). There have been reports of the anxiolytic effects of these four herbal medicines from other research teams, which are consistent with our conclusion. The well-known traditional Chinese formula Xiao Yao San, which is composed of eight herbs including Paeoniae Radix Alba, has exhibited therapeutic efficacy for mood disorders. Paeoniflorin extracted from this formula also exhibited anxiolytic-like effects against posttraumatic stress disorder [35]. In addition, the active components of Albiziae Flos have been experimentally shown to have an antidepressant effect, mainly via the regulation of monoaminergic neurotransmitters and cAMP signalling [36].

Our research used the CRS method to mimic the pathogenesis and pathophysiology of chronic stress-induced anxiety. We assessed this model from the perspective of the HPA axis and 5-HT system disorders. Stress-induced excessive activation of the HPA axis and downregulation of GR expression in the central nervous system are the key physiological features associated with long-term stress [6]. Our findings also confirm this conclusion. Specifically, JYHH treatment prevented this dysregulation by downregulating CRH, ACTH, and CORT and upregulating GR (Figure 4). Because many components of herbal medicines have hormone replacement effects, we speculate that this might be one of the possible mechanisms underlying this anxiety relief phenomenon [18]. Previous reports [37] have shown a close relationship between the HPA axis and anxiety, which is again consistent with our findings. For example, stress in rodents leads to an overactivated HPA axis characterised by increased CORT in plasma and higher CRH levels in the cortex [38]. The underexpression of GR in neural structures has a negative effect on behavioural changes and anxiolytic responsiveness in patients with chronic anxiety disorders [39].

Abnormal functioning of the HPA axis can cause serious physiological alterations in the central nervous system. In the hippocampus and PFC, dendritic shrinkage and spine loss were observed as a consequence of stress and CORT [40, 41]. Furthermore, CORT directly stimulates the release of excitatory amino acids and indirectly regulates both glutamate and *γ*-aminobutyric acid release [42]. In the current study, decreased concentrations of 5-HT and its metabolite (5-HIAA) were detected in the PFC of CRS-model rats, in which the high dose of JYHH prevented the 5-HT alteration (Figure 5). However, significant alterations were observed in 5-HT1AR and MAO-A mRNA and protein levels in both the PFC and hippocampus, and JYHH, especially at the high dose, and diazepam treatment exhibited satisfactory preventative effects (Figure 6). This indicates that CRS disturbs the 5-HT system mainly by modulating 5-HT1AR and MAO-A. Several therapeutic drugs for anxiety, such as selective monoamine neurotransmitter reuptake inhibitors, directly target 5-HT system dysfunctions [43]. Anxiolytic-like effects of 5-HT1AR agonists have been suggested [9]. A recent study indicated that acute CORT administration results in flattened hippocampal 5-HT responses, including a decrease in extracellular 5-HT levels and an increase in 5-HT reuptake efficiency [44]. Moreover, animals with 5-HT1AR deficiency show higher levels of anxiety, supporting this finding [9]. In addition, a Japanese team [16] reported that the herbal compound kamishoyosan could increase 5-HT1AR expression, which is similar to the effect of the Chinese herbal compound used in this study.

Because of the obvious differences in 5-HT1AR expression among the groups, we examined its downstream signalling pathway. 5-HT1AR is a typical G-protein coupled receptor that participates in the cAMP-PKA-CREB-BDNF pathway. Using ELISAs, western blotting, and RT-qPCR, the expression of relevant elements in this pathway was determined, and obvious differences were found in cAMP, PKA, p-CREB, and BDNF (Figures 7 and 8). Thus, the herbal compound was shown to have efficacy, especially in the high-dose group. BDNF is a growth factor that promotes neuronal proliferation and survival, neurodevelopment, and regeneration in the central nervous system [45]. BDNF is associated with the aetiopathology of mood disorders such as anxiety, depression, and stress-related disorders [46]. It can influence various cellular processes, including synaptic plasticity ranging from short- to long-lasting processes, on excitatory or inhibitory synapses and neuronal survival, and is regulated by glucocorticoids [46, 47]. Several anxiolytic and antidepressant drugs, including some herbs, were found to exhibit their efficacy through the regulation of BDNF [16, 22, 48]. Stress can induce abundant remodelling events in the brain that partially involve BDNF actions [49]. For example, consistent with our conclusion, decreased BDNF expression is associated with stress [50]. More importantly, chronic CORT exposure results in a decrease in BDNF expression, implicating the specific control of glucocorticoids on BDNF in the stress state [51]. CREB is initiated by the stimulation of several neurotransmitter receptors and is regulated by phosphorylation via cAMP-PKA [52]. A recent report showed that alterations in p-CREB/CREB and BDNF expression in stress-induced anxiety-like behaviours could be regulated by dammarane sapogenin treatment [53]. Another report indicated that resveratrol treatment ameliorates chronic unpredictable mild stress-induced depressive-like behaviour and cognitive deficits by upregulating CREB and brain BDNF [54].

**Figure 9:**
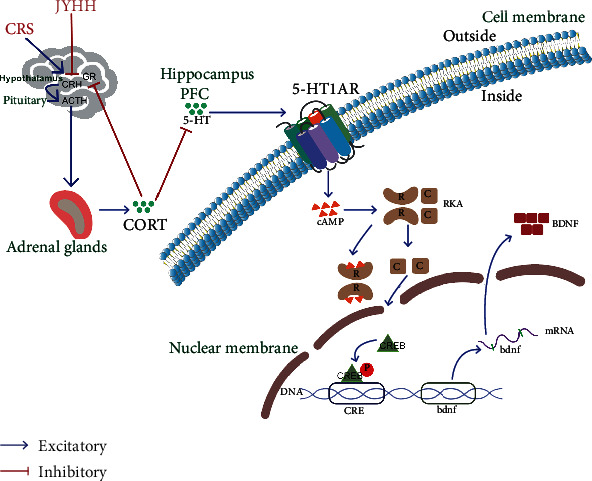
Schematic of the possible mechanism underlying the anxiolytic effect of the Jie-Yu-He-Huan capsule. The Chinese medicine formula Jie-Yu-He-Huan can ameliorate anxiety-like behaviours in rats exposed to chronic restraint stress by normalising the overactivated HPA axis function, ultimately regulating the 5-HT system, and finally targeting the cAMP-PKA-CREB-BDNF signalling pathway. HPA: hypothalamic–pituitary–adrenal; JYHH: Jie-Yu-He-Huan capsule; CRS: chronic restraint stress; CRH: corticotropin-releasing hormone; ACTH: adrenocorticotropic hormone; GR: glucocorticoid receptor; CORT: corticosterone; PFC: prefrontal cortex; 5-HT: 5-hydroxytryptamine; 5-HT1AR: 5-HT1A receptor; cAMP: cyclic adenosine monophosphate; PKA: protein kinase A; CREB: cAMP response element-binding protein; BDNF: brain-derived neurotrophic factor.

In this study, based on the behavioural anxiolytic drug effect of JYHH, we innovatively identified the underlying mechanism that can prevent abnormal alterations in cAMP-PKA-CREB-BDNF signalling. We offer a preliminary explanation of the possible mechanism underlying the effect of this compound from the perspectives of HPA axis function and the 5-HT system, as well as their close interrelationship. This provides relatively complete evidence to interpret the underlying mechanism as illustrated in Figure 9. Specifically, the Chinese medicine formula JYHH can ameliorate anxiety-like behaviours in rats exposed to CRS by normalising the overactivated HPA axis functions (downregulating the related hormones and upregulating GR), then regulating the 5-HT system (concentrations of 5-HT and 5-HIAA, and the expression of MAO-A and 5-HT1AR), and finally targeting the cAMP-PKA-CREB-BDNF signalling pathway (protein and mRNA expression of related factors).

To some extent, our study indicates that JYHH, which contains extracts from Paeoniae Radix Alba, Fructus Gardeniae, Albiziae Flos, and Moutan Cortex, is a potential therapeutic compound for stress-induced anxiety, which might provide a supplementary or preventive option for new anxiolytic drugs in the clinic. The main active components of JYHH are clear through HPLC analysis, which is of great scientific significance in revealing the mechanism. The results show the underlying target of JYHH might be related to the BDNF pathway, which plays a crucial role in neuroplasticity, neurodevelopment, and nerve regeneration [55], but this was rarely considered in previous research. A recent study showed stress gates the astrocytic energy reservoir, which could impair synaptic plasticity [56]. This might provide another perspective to help understand this process, which should be further explored in the future.

It is necessary to explore possible signal pathways in depth from the perspective of genetics. In our current study, signal pathway verification was only achieved on the whole animal level, and no cell experiment was carried out. The main consideration for not designing cell experiments is that the intervention drugs we use are traditional Chinese medicine compositions, and the ingredients are not all monomers. They cannot be directly added to the cell culture medium and can only be administered based on serum pharmacology [57]. However, this method is very limited and there are many controversies. In the next stage of our research, we plan to select the monomer components of the traditional Chinese medicine composition to verify the efficacy and design a series of cell experiments. Moreover, this study was limited to a specific signalling pathway and failed to explore other possible mechanisms related to stress-induced anxiety. We found that treating CRS with JYHH could influence the cAMP-PKA-CREB-BDNF pathway, which might be regulated by several factors. For example, dopamine and its receptor (another important G-protein coupled receptor) can also activate this signalling pathway [58]. Whether this and other possible systems are involved in stress-induced anxiety and drug efficacy remains to be studied. Moreover, JYHH showed dose-dependent characteristics, and the possible reasons were not explored. This suggests that we should perform more in-depth and detailed studies on this herbal compound and reveal the mechanisms from multiple perspectives. A more comprehensive chain of evidence from several aspects will help us to systematically understand drug activities. Furthermore, as anxiety and depression share a similar pathogenesis, whether this herbal compound is effective in improving depression-like symptoms remains to be further explored.

## 5. Conclusion

This study verified the anxiolytic drug efficacy of JYHH, a Chinese medicine formula, in a rat model of CRS-induced anxiety through several behavioural evaluations. We also provide evidence that the HPA axis, 5-HT system, and downstream cAMP-PKA-CREB-BDNF pathway could explain the underlying mechanism. Although additional investigation is still needed, our study provides initial support for a new supplementary or preventive option for anxiolytic drugs in the clinic and preliminarily illustrates the possible mechanism.

## Data Availability

The original contributions presented in the study are included in the article/supplementary material; further inquiries can be directed to the corresponding authors.
